# Multilayer Haze-Assisted Luminescent Solar Concentrators for Enhanced Photovoltaic Performance

**DOI:** 10.3390/ma18235422

**Published:** 2025-12-01

**Authors:** Jae-Jin Lee, Tae-Woong Moon, Dong-Ha Kim, Suk-Won Choi

**Affiliations:** Department of Advanced Materials Engineering for Information & Electronics, Kyung Hee University, Yongin 17104, Republic of Korea; jjking1443@naver.com (J.-J.L.); wecreatemind@khu.ac.kr (T.-W.M.); dongha0930@khu.ac.kr (D.-H.K.)

**Keywords:** polymer–liquid crystal composite, multilayer scattering, luminescent solar concentrator, building-integrated photovoltaics

## Abstract

Building-integrated photovoltaics (BIPVs) can benefit not only from transparent but also from opaque modules that maximize light capture. We present haze-assisted luminescent solar concentrators (HALSCs) that integrate scattering and luminescence in multilayer designs. Polymer–liquid crystal composites with embedded dyes form micron-scale domains that act as broadband Mie scattering centers, while the dye provides spectral conversion. Monte Carlo ray-tracing simulations and experiments reveal that edge-emitted intensity increases with haze thickness but saturates beyond a threshold; segmenting the same thickness into multiple thinner layers enables repeated scattering, markedly enhancing side-guided emission. When coupled with crystalline silicon solar cells, multilayer HALSCs converted this optical advantage into enhanced photocurrent, with triple-layer devices nearly doubling output relative to transparent controls. These findings establish opacity–luminescence coupling and multilayer haze engineering as effective design principles, positioning HALSCs as practical platforms for advanced BIPVs and optical energy-management systems.

## 1. Introduction

Luminescent solar concentrators (LSCs) are widely regarded as promising candidates for building-integrated photovoltaics (BIPVs) and solar-window technologies [1,2,3]. In a typical LSC, luminescent dyes or quantum dots dispersed in a transparent host absorb incident sunlight and re-emit it at longer wavelengths. The re-emitted photons are then guided by total internal reflection (TIR) toward photovoltaic (PV) cells positioned at the device edges [4,5,6,7,8]. Owing to this simple yet effective mechanism, LSCs have attracted considerable interest for their ability to convert large-area surfaces into energy-harvesting platforms [9,10].

Most prior studies have focused on transparent or semi-transparent LSCs, primarily to support window-integrated applications [11,12,13]. However, transparency is not always essential for BIPV installations. In many architectural contexts, non-transparent modules can be equally practical—or even preferable—when maximizing solar capture is more important than maintaining optical clarity. This consideration motivates the exploration of concentrator designs that are intentionally opaque.

In this work, we introduce haze-assisted LSCs (HALSCs) as inherently opaque devices. Unlike conventional transparent LSCs, HALSCs incorporate scattering-induced haze within the luminescent medium, ensuring constant opacity. By treating opacity as a functional design parameter, HALSCs combine scattering and luminescence to enhance light harvesting beyond the limits of re-emission alone.

The key novelty of this study is that the haze layers actively promote TIR by repeatedly re-scattering emitted photons, thereby strengthening lateral photon confinement within a multilayer HALSC architecture. To realize this concept, we fabricated microphase-separated haze films via UV-induced phase separation of nematic liquid crystals (NLCs) and photocurable polymers. The resulting micron-scale domains serve as broadband Mie-scattering centers that redistribute sunlight into TIR-supported guided modes, while embedded dye molecules provide absorption and re-emission, extending the usable spectral range. Together, these effects enhance photon confinement and waveguiding efficiency. We additionally investigated multilayer scattering architectures in which the haze–dye films were divided into stacked segments. This configuration offers additional opportunities for photons that are not initially trapped to undergo repeated scattering and redirection, thereby increasing their probability of being retained within the waveguide.

Overall, our results demonstrate that HALSCs expand the design space of LSCs by combining opacity, scattering–luminescence coupling, and multilayer structuring. This practical approach highlights new opportunities for BIPV applications where optical transparency is not required.

## 2. Materials and Methods

To prepare films that combine luminescent and scattering functionalities, we fabricated a polymer–NLC composite layer containing a fluorescent dye. A UV-curable prepolymer (NOA88, Norland Products, Jamesburg, NJ, USA) and a nematic liquid crystal (HTW109100-100, HCCH, Nanjing, China) were mixed in a 7:3 weight ratio, followed by the addition of 0.5 wt% Coumarin 343 (Sigma-Aldrich, St. Louis, MO, USA). Upon UV curing, micron-scale phase separation produced NLC-rich droplets dispersed within the polymer matrix, forming a polymer-dispersed liquid crystal (PDLC) structure [14]. A histogram of the LC droplet size distribution for the PDLC films fabricated in this work is shown in Figure S1, with a representative polarized optical micrograph provided in the inset. The droplets exhibit diameters below 3 μm, with an average of 1.43 μm. The refractive-index mismatch between the polymer and NLC phases causes the droplets to function as Mie scattering centers, redirecting incident light into waveguiding angles, while the dye molecules absorb UV light and re-emit in the visible range.

Coumarin 343 was selected as the luminescent dye for three reasons: (i) its high fluorescence quantum yield enables efficient photon conversion, (ii) its broad Stokes shift reduces reabsorption and concentration quenching [15], and (iii) its absorption–emission profile (UV–blue absorption and green emission) aligns well with the spectral response of crystalline silicon (Si) solar cells. Together, these characteristics provide favorable spectral matching and support effective photon confinement, contributing to enhanced photovoltaic performance. The absorbance and photoluminescence spectra of Coumarin 343 are presented in Figure S2. The selected dye and polymer matrix were chosen for their well-established optical properties, enabling a clear evaluation of the structural design concept without the added complexity of material-specific optimization.

To study structural effects, we designed multilayer scattering architectures in which the total scattering thickness was fixed at 100 μm while the number of dye-doped layers was varied. Three HALSC configurations were prepared: HALSC-1 (100 μm), HALSC-2 (2 × 50 μm), and HALSC-3 (3 × 30 μm). Because 33 μm spacers were not readily available, the triple-layer samples were fabricated using 30 μm spacers instead. Although this did not yield an exact total of 100 μm, the overall scattering thickness remained very close to the intended value. The fabrication process is illustrated in Figure S3.

**Figure 1:**
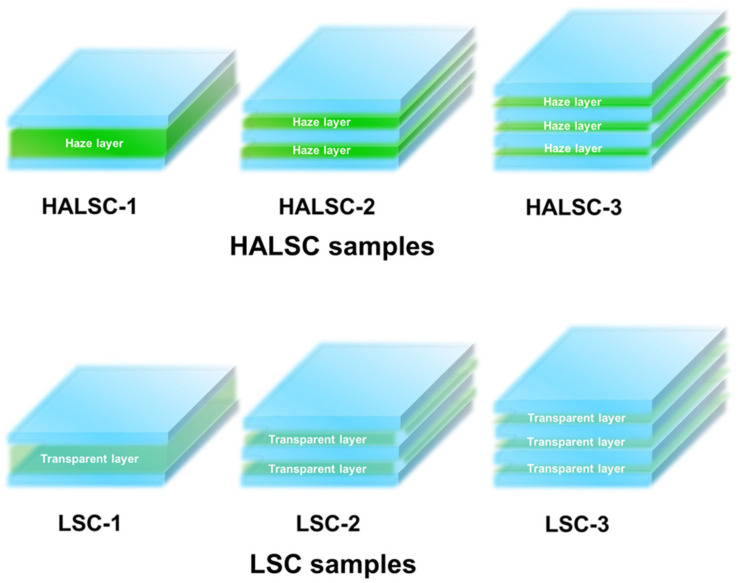
Schematic illustration of the multilayer HALSC and LSC architectures.

For comparison, reference LSC samples with identical total thicknesses but without scattering domains were fabricated: LSC-1 (100 μm transparent), LSC-2 (2 × 50 μm transparent), and LSC-3 (3 × 30 μm transparent). These transparent reference layers were prepared by adding 0.5 wt% Coumarin 343 directly to the UV-curable prepolymer (NOA88). The overall sample architectures are summarized in Figure 1.

In this study, the HALSC samples were not configured for electric-field application. Nevertheless, if a vertical electric field were applied, the PDLC scattering state could be switched to a transparent state. In such a case, a HALSC sample under an applied field would effectively function as the LSC samples fabricated in this work.

## 3. Results and Discussion

### 3.1. Thickness- and Layer-Dependent Edge Emission

**Figure 2:**
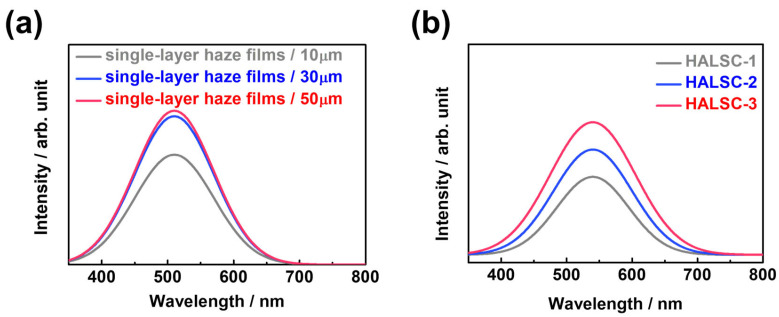
(**a**) Monte Carlo ray-tracing simulation showing the thickness-dependent guided light intensity for single-layer haze films (10, 30, 50 µm). (**b**) Simulation results for one-, two-, and three-layer HALSC structures with identical total thickness (100 µm).

First, the thickness-dependent behavior of single-layer haze films (10, 30, and 50 μm) was investigated through Monte Carlo ray-tracing simulations [16,17,18] (Figure 2a). The guided-light intensity increased with haze-layer thickness but saturated beyond ~30 μm, indicating that once a sufficient scattering volume is reached, additional thickness provides little further improvement. In the simulations, the haze film was modeled as a polymer matrix containing randomly distributed NLC-rich domains with diameters of 1–3 μm, satisfying the Mie scattering condition. The PDLC layer was sandwiched between glass substrates, and because the refractive index mismatch between PDLC and glass was negligible (n_PDLC_ ≈ n_glass_), total internal reflection (TIR) occurred only at the outer glass–air interfaces (n_glass_ ≈ 1.52 vs. n_air_ ≈ 1.0; critical angle θ_c_ ≈ 41.8°). Photons were tracked through scattering, refraction, and reflection events using the open-source Python-based Monte Carlo ray-tracing package pvtrace 2.0.1, and those meeting the TIR criterion at the glass–air boundary were counted as guided light.

Next, multilayer films with a constant total thickness (~100 μm) but varying segmentation (one, two, or three layers; Figure 2b) were examined. Both experiments and simulations revealed stepwise enhancement as the number of layers increased. Notably, HALSC-3 (three 30 μm layers) exhibited significantly higher side-guided emission than HALSC-1 (one 100 μm layer) or HALSC-2 (two 50 μm layers), demonstrating the superiority of segmented architectures. This improvement does not originate from additional TIR at PDLC–glass interfaces, since the refractive index mismatch between PDLC and glass is negligible and no new TIR sites are introduced. Instead, the enhancement is attributed to repeated re-scattering events within the PDLC layers, which provide additional opportunities for photons to be redirected above the critical angle at the outer glass–air interface, thereby increasing the probability of waveguiding and enhancing lateral light confinement. Further details on the simulations of thickness- and multilayer-dependent behaviors are provided in the Supporting Information.

Taken together, these findings indicate that the multilayer HALSC architecture provides additional opportunities for photons to undergo repeated scattering, increasing the likelihood that they are redirected above the critical angle and subsequently confined by total internal reflection at the glass–air interfaces. This repeated-scattering mechanism is therefore central to the enhanced lateral photon collection observed in the multilayer structures. Even if some degree of dye reabsorption were present, the enhancement in multilayer HALSCs would still be dominated by repeated scattering events within the segmented layers, which more effectively redirect photons into waveguiding modes. Thus, the improved performance arises primarily from scattering-assisted photon management rather than from changes in reabsorption.

### 3.2. Comparison of Multilayer LSC and HALSC

**Figure 3:**
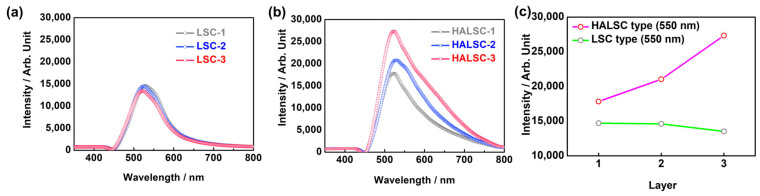
(**a**) Edge-emitted light intensity of LSC-1, LSC-2, and LSC-3. (**b**) Edge-emitted light intensity of HALSC-1, HALSC-2, and HALSC-3. (**c**) Comparison of edge-emitted light intensity at 550 nm between LSC and HALSC samples.

The performance of conventional LSCs and HALSCs was experimentally compared using one-, two-, and three-layer structures. Figure 3a,b show the edge-emitted photon intensities measured under AM1.5G illumination using an integrating sphere and spectrometer. As shown in Figure 3a, the edge-emitted intensities of LSC-1, LSC-2, and LSC-3 remain nearly unchanged even when the luminescent layer is divided into multiple segments. Because the transparent LSC films contain no scattering domains, segmentation does not introduce additional redirection events or alter photon trajectories; consequently, all segmented LSC configurations exhibit essentially identical edge-guided emission.

In contrast, HALSCs (Figure 3b) display a clear increase in side-guided intensity with each additional haze layer. This behavior indicates that the scattering domains not only redirect photons into guided modes but also provide repeated opportunities for re-scattering when arranged in multiple layers. As supported by the simulation results, these repeated scattering events enhance optical confinement and increase the likelihood of TIR at the outer glass–air interfaces. Figure 3c summarizes the guided-light intensity at 550 nm, showing that HALSC-3 achieves nearly double the side-emitted intensity of LSC-3. These findings highlight that the presence of scattering domains, combined with multilayer structuring, is essential for maximizing lateral photon confinement.

Although various LSC concepts have been reported, their optical architectures and measurement conditions differ substantially, preventing standardized quantitative comparison. Therefore, the enhancement demonstrated in this work is assessed relative to transparent reference structures fabricated and measured under identical conditions, enabling the contribution of the haze-assisted multilayer design to be clearly isolated.

In addition to the layer-dependent performance differences, we note that the actual haze-layer thickness of HALSC-3 was approximately 90 μm rather than the target 100 μm due to spacer availability. However, both values fall within the saturation regime identified in Figure 2a, and this small deviation is therefore not expected to affect the photon-guiding behavior or the conclusions regarding multilayer enhancement.

### 3.3. Photovoltaic Performance

**Figure 4:**
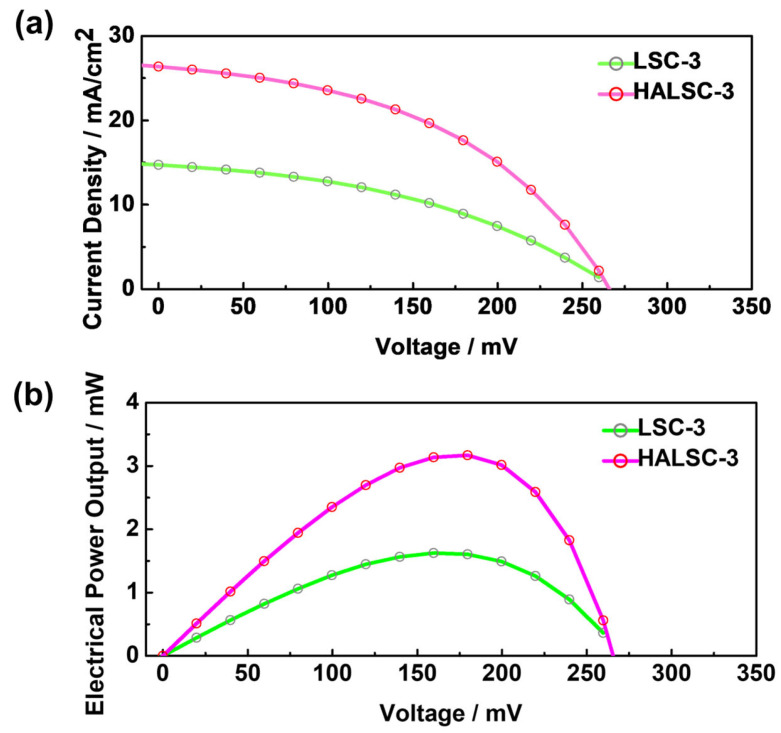
(**a**) Current–voltage (J–V) characteristics of the Si cell coupled with LSC-3 and HALSC-3. (**b**) Electrical power output (P) of the Si cell coupled with LSC-3 and HALSC-3 as a function of voltage.

To evaluate photovoltaic performance, a commercial crystalline Si solar cell was attached to one edge of representative LSC-3 and HALSC-3 samples, while the remaining edges were covered with black tape to suppress unintended light leakage. Current–voltage (J–V) measurements were performed under AM1.5G illumination using a commercial solar simulator and a source meter (Keithley 2400, Keithley Instruments, Solon, OH, USA). Figure 4a presents the J–V characteristics of the Si cell coupled to LSC-3 and HALSC-3. The short-circuit current density of the cell with LSC-3 was 12.74 mA, whereas that with HALSC-3 was 26.36 mA. The corresponding electrical power output (P), obtained by multiplying the measured current and voltage, is shown in Figure 4b. The maximum power of the Si cell coupled to LSC-3 and HALSC-3 was 1.62 mW and 3.17 mW, respectively, indicating that the HALSC-3 device delivered nearly twice the output power of its LSC counterpart. This enhancement is attributed to the substantially stronger side-emitted intensity of HALSC-3 relative to LSC-3. These results provide preliminary validation that the multilayer haze architecture effectively recaptures and redirects light, demonstrating the superior performance of HALSCs over conventional LSCs. Therefore, for BIPV applications where transparency is not required, the HALSC architecture represents a highly promising approach.

## 4. Conclusions

In this study, we demonstrated HALSCs that leverage opacity and multilayer scattering–luminescence coupling to enhance photon confinement and photovoltaic output. Unlike conventional LSCs, the multilayer HALSCs exhibited progressive improvements in both edge emission and photocurrent. This work establishes opacity as a functional design parameter and highlights multilayer haze engineering as an effective light-management strategy for BIPV applications in which transparency is not required. Future optimization through spectral tuning, advanced layer architecture, and integration with adaptive materials may further extend the potential of HALSCs in solar energy and broader photonic technologies.

## Data Availability

The original contributions presented in this study are included in the article/Supplementary Material. Further inquiries can be directed to the corresponding author.

## Supplementary Materials

The following supporting information can be downloaded at: https://www.mdpi.com/article/10.3390/ma18235422/s1, Figure S1: Histogram of LC droplet size distribution; Figure S2: Absorbance and photoluminescence spectra of Coumarin 343; Figure S3: Illustration of fabrication process of multilayer HALSC samples; Details of Monte Carlo Ray-Tracing Simulation for Figure 2a,b.
