# Supplementary material for: Multilayer Haze-Assisted Luminescent Solar Concentrators for Enhanced Photovoltaic Performance
Source: Materials (Basel). 2025 Dec 1;18(23):5422. doi: 10.3390/ma18235422 (PMC12692778; doi:10.3390/ma18235422)
Supplement: Supplementary file 1 [file materials-18-05422-s001.zip › materials-3975967-supplementary.pdf]

Supplementary Materials

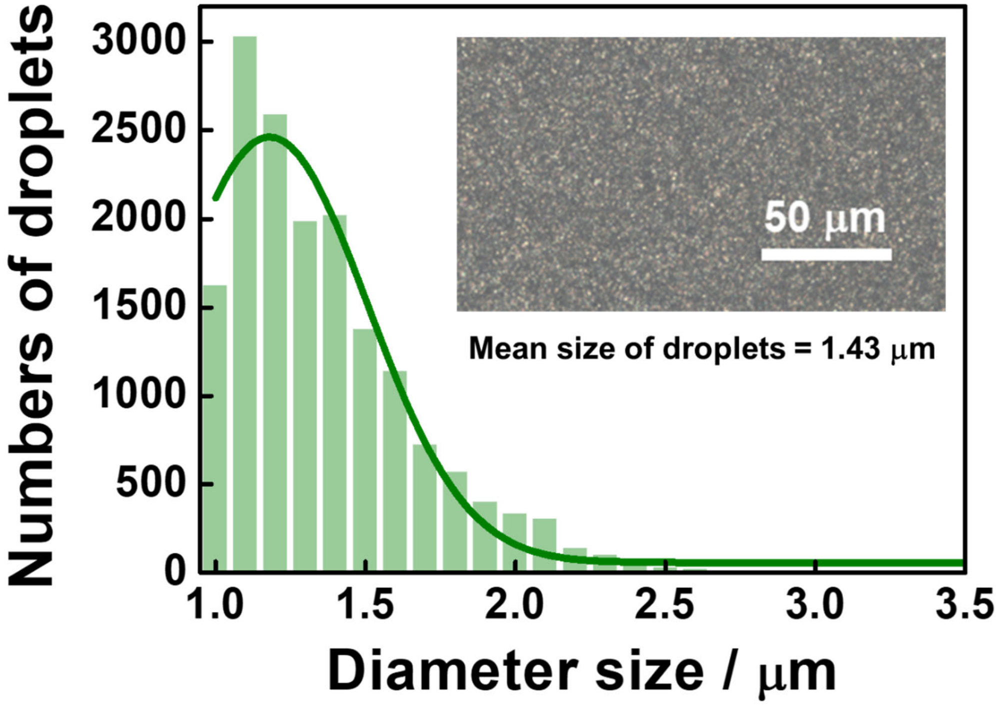

Figure S1. Histogram of LC droplet size distribution in the PDLC films fabricated in this work. A representative polarized optical micrograph is shown in the inset.

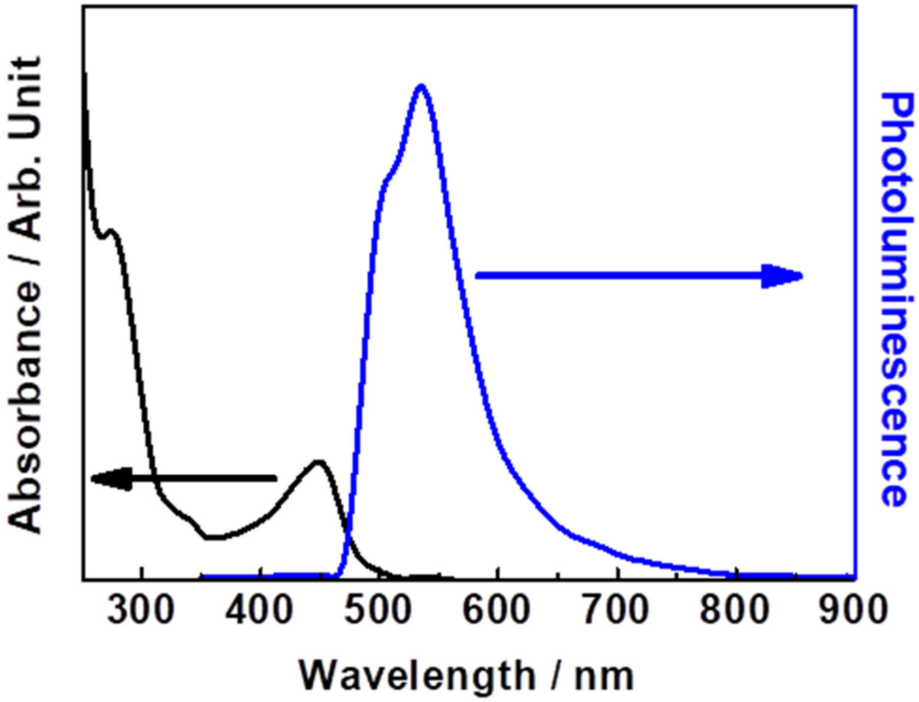

Figure S2. Absorbance and photoluminescence spectra of Coumarin 343.

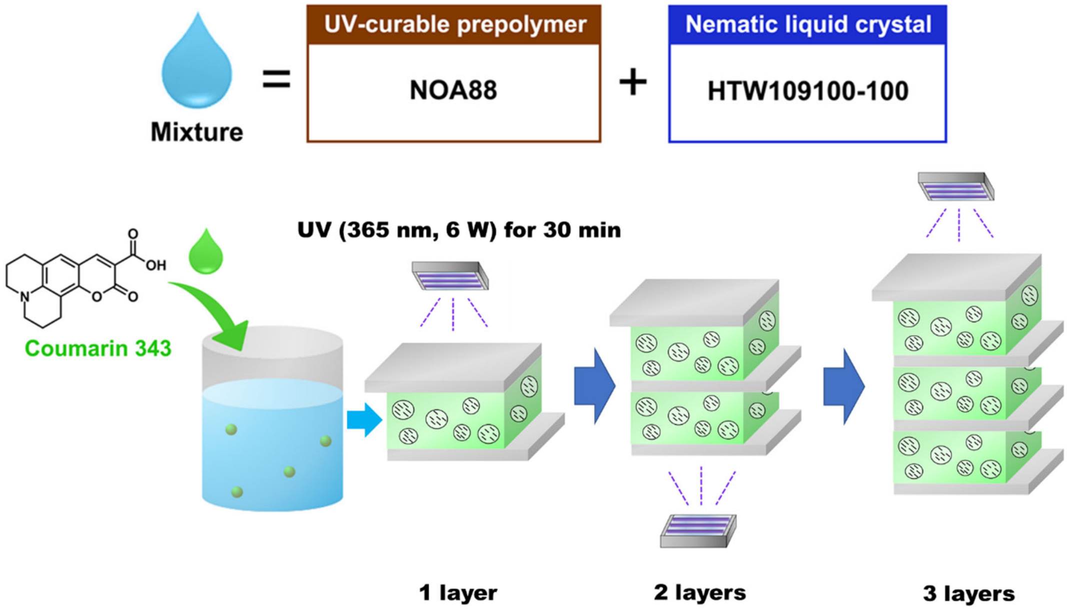

Figure S3. Illustration of fabrication process of multilayer HALSC samples.

Monte Carlo Ray-Tracing Procedure for Figure 2a

Monte Carlo ray-tracing was employed to model photon transport in single-layer haze films of varying thicknesses (10, 30, and 50 μm). The simulated structure consisted of a haze layer sandwiched between glass substrates (n_{glass} = 1.52), with an effective refractive index of n_{haze} ≈ 1.50 for the polymer/NLC mixture. The external medium was air (n_{air} = 1.00), giving a critical TIR angle of $\theta_c \approx 41.8°$ at the glass–air interface.

S1.1 Photon Launching and Tracking

Photon packets (typically 10^6 per simulation) were launched under normal incidence from the top surface. Each photon was propagated through a sequence of scattering, refraction, and reflection events until it either:

1. escaped through an external boundary,

2. entered a TIR-guided mode ($\theta_i > \theta_c$), or

3. exceeded the maximum scattering count (10,000 events).

Photon trajectories were updated according to Snell's law and Fresnel coefficients at each interface.

S1.2 Scattering Model

The haze layer was treated as a continuous medium containing randomly distributed NLC-rich

domains representing Mie scattering centers. Photon free paths were sampled from an exponential distribution using a scattering mean free path l_s chosen to reproduce the experimentally measured haze.

Scattering angles were drawn from the Henyey–Greenstein phase function:

$$P(\cos\theta) = (1/2) * (1 - g^2) / (1 + g^2 - 2g \cos\theta)^{3/2},$$

with anisotropy factors g = 0.7–0.85, consistent with forward-peaked scattering from micron-sized domains. Azimuthal angles were uniformly distributed over 0–2π.

S1.3 Reflection, Refraction, and TIR Conditions

At glass–haze and glass–air boundaries, Fresnel equations were used to determine reflection (R) and transmission (T) probabilities.

For the glass–air interface:

- photons with $\theta_i > \theta_c$ were classified as TIR-guided,

- photons with $\theta_i < \theta_c$ escaped.

Guided photons were counted to estimate the guided-light fraction.

S1.4 Statistical Convergence

Simulations were repeated three times to ensure statistical stability. The variation in guided-light intensity among runs was <1%. Results were normalized to the total number of launched photons.

S1.5 Summary of Results

The simulation results reproduced the experimental trend:

- Guided-light intensity increased markedly from 10 to 30 μm,
- but showed minimal further increase at 50 μm, indicating saturation of the scattering volume.

Thus, once the haze layer is sufficiently thick for photons to experience multiple scattering events, additional thickness provides only marginal improvement in coupling into TIR-supported guided modes.

Details of Monte Carlo Ray-Tracing Simulation for Figure 2b

Monte Carlo ray-tracing was extended to evaluate photon transport in multilayer haze films with a fixed total thickness of 100 μm. Three configurations were simulated:

1. Single-layer: 100 μm

2. Two-layer: 50 μm + 50 μm

3. Three-layer: 33 μm + 33 μm + 33 μm (experimentally approximated as 3 × 30 μm)

In all cases, the haze structure was sandwiched between glass substrates (n_{glass} = 1.52). The effective refractive index of the haze region was set to n_{haze} ≈ 1.50, and the external medium was air (n_{air} = 1.00), giving a critical angle of θ_c ≈ 41.8° for total internal reflection (TIR) at the glass–air boundary.

S2.1 Photon Launching and Boundary Conditions

Photon packets (typically 10^6 per simulation) were launched under normal incidence from the top surface. Each photon was traced until it:

- exited the structure,

- reached the glass–air interface with $\theta_i > \theta_c$ (classified as TIR-guided), or

- exceeded the maximum number of allowed scattering events (10,000).

Snell's law and Fresnel coefficients for s- and p-polarized light (averaged) were applied at all

interfaces.

S2.2 Scattering Model

The haze layer was modeled as a continuous medium containing randomly distributed NLC-rich domains acting as Mie scattering centers.

For each sub-layer:

- droplet positions were independently generated;

- the scattering mean free path l_s was selected to reproduce experimentally measured haze values;

- scattering angles were sampled from the Henyey–Greenstein function with anisotropy factor $g = 0.7–0.85$;

- azimuthal angles were uniformly sampled between 0 and 2π.

This approach ensures that segmentation alters the distribution of scattering events without altering their statistical properties.

S2.3 Multilayer Interfaces

Internal interfaces between haze layers were treated as optically continuous because:

- $|n_{haze} - n_{glass}| < 0.02$,

- Fresnel reflections at these boundaries are <0.1%.

Accordingly, no internal TIR or additional reflections arise within the multilayer stack. Segmentation therefore influences guiding only by modifying where scattering occurs, not by index contrast effects.

S2.4 Effect of Layer Segmentation

Dividing the total haze thickness into multiple layers increases the frequency with which photons re-enter scattering-rich regions. This enhances the likelihood of angular redistribution into trajectories above θ_c, thereby increasing the number of photons that become trapped by TIR at the outer glass–air boundary.

S2.5 Summary of Simulation Results

The simulation results reproduce the experimental trend:

- guided-light intensity increases stepwise as the number of layers increases,

- with the three-layer configuration showing the highest confinement efficiency.

These results confirm that the benefit of segmentation arises from scattering-assisted angular redistribution, rather than from refractive-index effects at internal interfaces.
